# Interferon regulatory factor 1 (IRF1) and anti-pathogen innate immune responses

**DOI:** 10.1371/journal.ppat.1009220

**Published:** 2021-01-21

**Authors:** Hui Feng, Yi-Bing Zhang, Jian-Fang Gui, Stanley M. Lemon, Daisuke Yamane

**Affiliations:** 1 Lineberger Comprehensive Cancer Center, The University of North Carolina at Chapel Hill, Chapel Hill, North Carolina, United States of America; 2 Hebei Province Cangzhou Hospital of Integrated Traditional Chinese and Western Medicine, Cangzhou, Hebei, China; 3 State Key Laboratory of Freshwater Ecology and Biotechnology, Institute of Hydrobiology, Chinese Academy of Sciences, Wuhan, Hubei, China; 4 Department of Medicine, The University of North Carolina at Chapel Hill, Chapel Hill, North Carolina, United States of America; 5 Department of Microbiology & Immunology, The University of North Carolina at Chapel Hill, Chapel Hill, North Carolina, United States of America; 6 Department of Diseases and Infection, Tokyo Metropolitan Institute of Medical Science, Setagaya-ku, Tokyo, Japan; University of Queensland, AUSTRALIA

## Abstract

The eponymous member of the interferon regulatory factor (IRF) family, IRF1, was originally identified as a nuclear factor that binds and activates the promoters of type I interferon genes. However, subsequent studies using genetic knockouts or RNAi-mediated depletion of IRF1 provide a much broader view, linking IRF1 to a wide range of functions in protection against invading pathogens. Conserved throughout vertebrate evolution, IRF1 has been shown in recent years to mediate constitutive as well as inducible host defenses against a variety of viruses. Fine-tuning of these ancient IRF1-mediated host defenses, and countering strategies by pathogens to disarm IRF1, play crucial roles in pathogenesis and determining the outcome of infection.

## Introduction

**Fig 1:**
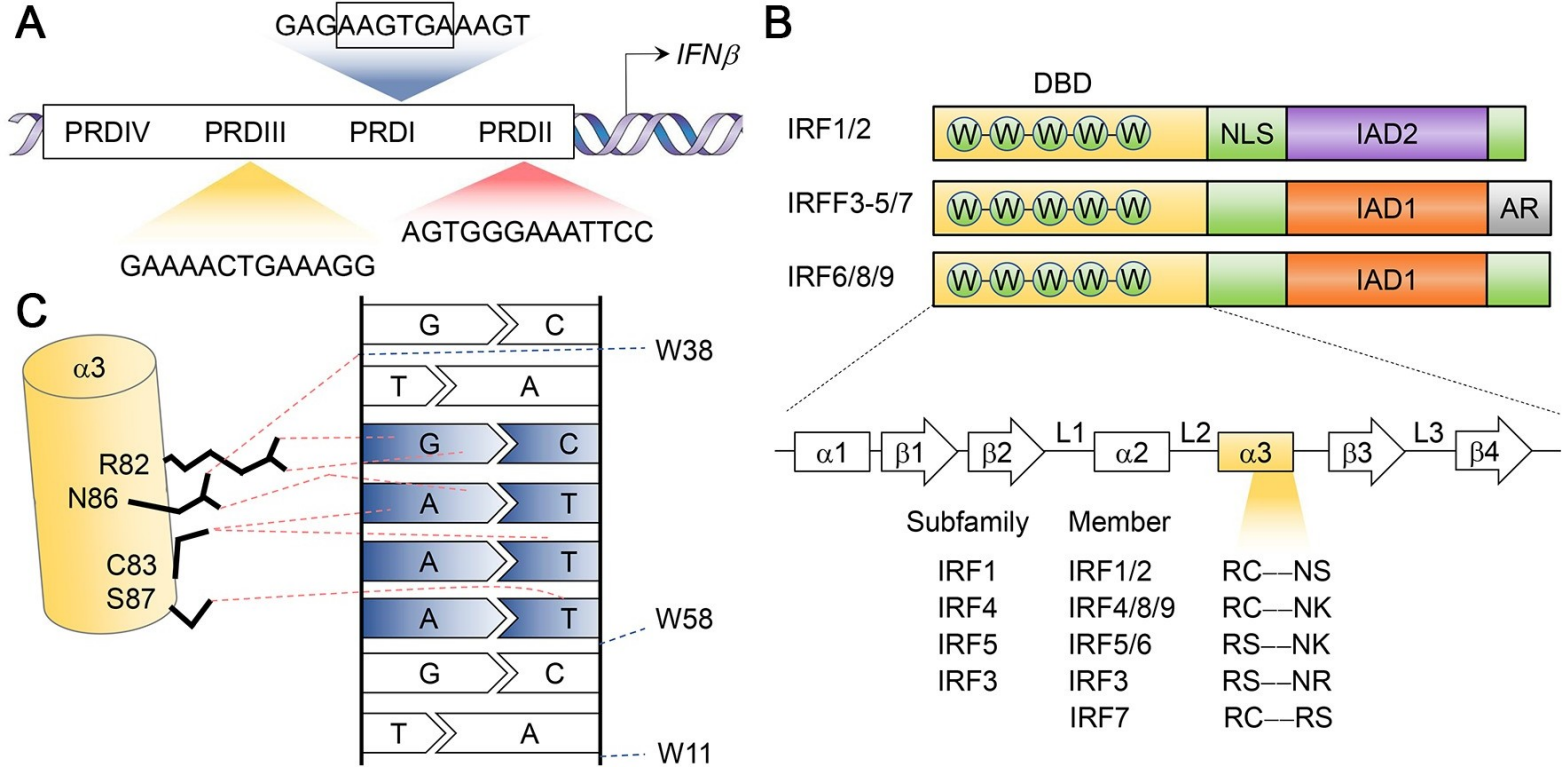
The discovery and recognition mechanism of IRF1. **(A)** The four positive regulatory domains (PRDs) within IFNβ promoter (nucleotides -105 to +19 relative to the transcriptional start site). **(B)** Key features of the IRF family members. DBD, DNA binding domain; IAD, IRF-association domain; AR, auto-inhibitory region; α1–3, α helices 1–3; β1–4, β sheets 1–4; L1-3, loops 1–3. **(C)** The detailed structural study of DNA-binding by IRF proteins involved the DBD of IRF1. Trp11, Trp38 and Trp58 of IRF1 strategically straddle the major groove of the ‘GAAA’ segment of the DNA, forming hydrogen bonds and van der Waals contacts with the sugar-phosphate backbone (blue dashed lines). Arg82, Cys83, Asn86 and Ser87, all within the α3 recognition helix, mediate direct contacts between the DBD and GAAA (red dashed lines). With high structural similarities of the DBD, IRF family members recognize consensus ‘AANNGAAA’ sequence, yet they display slightly different binding specificities [120].

Innate immunity provides a critical biological barrier protecting the host from invading pathogens. Given its crucial role as a first line of defense, multiple innate immune mechanisms are broadly conserved among the vertebrates. Cells engaging in such responses undergo multi-layered signal transduction originating at pattern recognition receptors (PRRs) that sense pathogen-associated molecular patterns (PAMPs), leading to the rapid induction of gene expression programs that broadly target invading viruses and other pathogens. A hallmark feature of this response is the production of interferons (IFNs). Although the anti-viral function of IFNs was recognized as early as 1957 [1], the genetic elements (positive regulatory domains, PRDs) in host DNA responsible for the induction of transcription of the gene encoding IFNβ (*IFNB1*), a prototypical type I IFN, were not unveiled until the 1980s (**Fig 1A**) [2]. These seminal findings from the Maniatis laboratory and others laid the foundation for understanding the molecular basis of the transcriptional regulation of IFNs.

In 1988, IFN-regulatory factor 1 (IRF1), the first member of the IRF family to be recognized, was identified by the Taniguchi laboratory in crude nuclear extracts of Newcastle disease virus (NDV)-infected mouse L929 cells using DNA affinity column chromatography [3]. Despite elegant proof of its ability to regulate *IFNB1* transcription, the biological importance of IRF1 in inducing IFN responses was questioned not long after its discovery [4]. Indeed, NDV was shown to induce normal expression of type I IFNs in embryonic fibroblasts, embryonic stem cells, and even in mice depleted of IRF1 expression [5,6], giving IRF1 relatively short-lived prominence among blossoming studies of the IFN response. Other members of the IRF family, particularly IRF3 and IRF7, emerged as critical regulators of IFN transcription by the first decade of the 21^st^ century. In parallel, multiple studies found IRF1 to be associated with tumor suppression, natural killer (NK) and T cell development, and B cell biology, changing the focus of the field [7–9]. However, over the past decade, previously unappreciated aspects of the role of IRF1 in innate immunity have come to light. Here, we review recent progress in understanding how IRF1 contributes to innate immunity against pathogens, particularly RNA viruses, focusing on its evolution, mechanisms of signal transduction, and multi-scale role in immune defense.

### Transcriptional activity of IRF1

Ubiquitously expressed in human cells at low basal levels [10], the *IRF1* gene is highly responsive to a variety of stimuli, including IFNs and the pro-inflammatory nuclear factor kappa-B (NF-κB) [11–13]. Thus, *IRF1* fits the typical profile of an “IFN-stimulated gene” (ISG). Both *IRF1* mRNA transcripts and the IRF1 protein itself are short-lived [14], allowing for rapid, dynamic regulation in response to infection. However, low basal levels of IRF1 are present in the nucleus where it maintains constitutive expression of a variety of host defense genes [15], as described in detail below. Whether induced in response to infection or basally expressed, the impact of IRF1 on innate immunity stems from its capacity to drive gene expression.

The transcriptional activity of IRF1 depends on its 115 amino-acid N-terminal DNA binding domain (DBD) which contains five tryptophan residues, spaced in a characteristic fashion shared by all 9 mammalian members of the IRF family (**Fig 1B**) [16]. Immediately downstream of the DBD is a nuclear localization signal (NLS, amino acid residues 117–141) that facilitates IRF1 nuclear residency [17]. This is followed by a C-terminal IRF-association domain (IAD2, amino acid residues 200–262), unique to the IRF1 subfamily, that mediates interactions with other transcriptional cofactors, such as IRF8 (*a*.*k*.*a*. ICSBP) [18]. The concerted activities of these three functional domains determines the pathophysiologic impact of IRF1 during infection.

In early studies, IRF1 was found to bind several hexanucleotide units within the IFNβ promoter [3,19]. An ‘AAGTGA’ motif located within PRDI was particularly prominent, with the binding affinity of IRF1 correlating with the number of ‘AAGTGA’ repeat units. Detailed footprint and mutational analyses suggested that the actual recognition sequence of the DBD was ‘GTGAAA’, leading to the positive regulatory sequences that contain such hexanucleotide units being referred to as IRF-binding elements (IRF-E) [20]. Subsequent crystallographic studies of an IRF1 and PRDI complex provided a more complete understanding of IRF1 recognition (**Fig 1C**) [21]. It came as no surprise when the conserved tryptophan cluster within the DBD was found to be critical in recognition of the core ‘GAAA’ sequence. Among the five tryptophan residues, three (W11, W38 and W58) fix a helix-turn-helix motif within the DBD onto the major groove of the DNA, forming contacts with the sugar-phosphate backbone and allowing direct contacts to the ‘GAAA’ bases by four amino acids (R82, C83, N86 and S87) in a third, obliquely-angled recognition helix.

### Evolutionary conservation of IRF1

**Fig 2:**
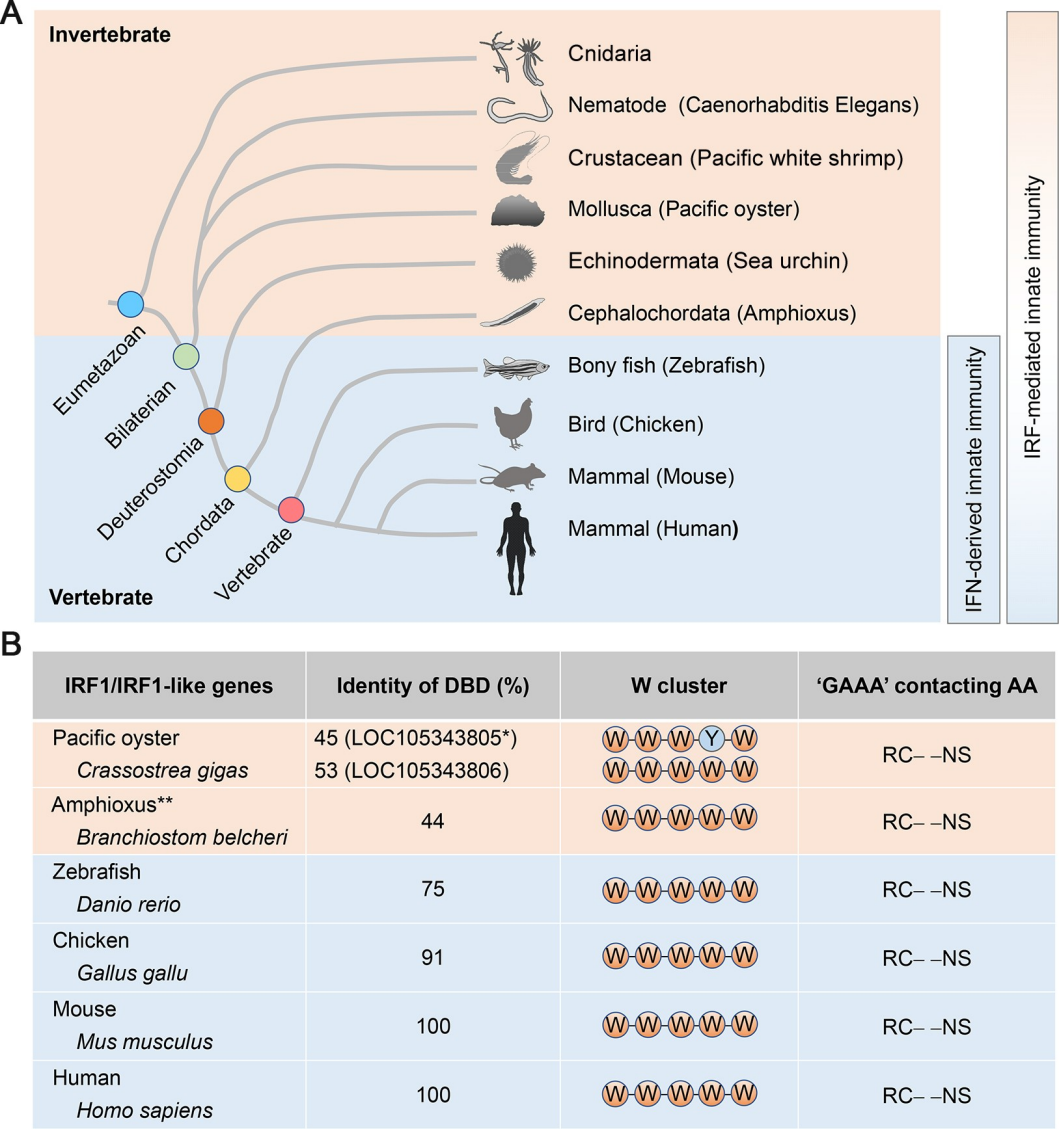
Evolutionary conservation of IRF1. **(A)** Phylogenetic occurrence of IRF- and IFN-dependent innate immune response. The selection of individual species is based on literature indicating certain effects and is not intended to be comprehensive. In cnidarians and bilaterians, which diverged from the common eumetazoan ancestor ~600–630 million years ago, IRFs are proposed to be separated into IRF1 and IRF4 subfamilies. **(B)** Evolutionary comparison of vertebrate IRF1 and invertebrate IRF1-like genes within the DBD. *, *C*. *gigas* ‘IRF1’ gene that has been reported. Three other predicted transcript variants of Pacific oyster IRF1 exist (LOC105343806; NCBI Accession numbers: XM_011451290.2 for X1, XM_011451291.2 for X2 and XM_011451292.2 for X3). The predicted amino acid sequence shares 53% identity with *H*. *sapiens* IRF1 at the DBD, and contains all five tryptophans as well as the conserved GAAA-contacting residues. LOC105343805 thus might not represent *bona fide C*. *gigas* IRF1. **, *B*. *belcheri* ‘IRF1’ does not present a rigid one-to-one ortholog relationship with vertebrate IRF1. Rather, it is linked to the vertebrate IRF1 subfamily based on phylogenetic analysis.

Unlike IFNs, IRFs (or at least IRF-like genes with homology to mammalian IRFs) exist in a wide swath of invertebrates (**Fig 2A**) [22,23]. Although incompletely characterized in invertebrates, phylogenetic analysis suggests that primordial IRFs diverged into IRF1 and IRF4 subfamilies as early as in cnidarians and bilaterians [24], implying that IRF1 represents one of the most ancient components of innate immunity. Although the evolutionary origins of IRF1 remain unclear, IRF1 orthologues have been identified in widely-divergent vertebrate species, including fish, the phylogenetically most distant vertebrates possessing an IFN system [25]. Notably, early studies of zebrafish IRF1 recapitulated struggles in the identification of its mammalian counterparts, as IRF11, a novel IRF unique to teleost fish with close homology to vertebrate IRF1, was discovered prior to the authentic zebrafish IRF1 gene, leading to confusion over its proper annotation [26]. Recently, IRF1-like genes have also been identified in the amphioxus *Branchiostoma belcheri* and Pacific oyster *Crassostrea gigas* (**Fig 2B**) [27,28].

Although invertebrate IRF1-like genes with homology to mammalian IRF1 share far less identity with human IRF1 than vertebrate orthologues, the elements mediating recognition of the GAAA sequence are well preserved (**Fig 2B**). It is therefore not surprising that the overarching function of IRF1/IRF1-like genes in activating specific regulatory elements is evolutionarily conserved [26]. This notion is strengthened by the evolutionally conserved nature of the vaccinia host range gene C7L, homologues of which from a variety of poxviruses counteract IRF1-induced antiviral activities [29]. Equally remarkable, a recent study of ‘interferomes’ identified IRF1 as one of the key transcriptional factors included in a core group of 62 ISGs shared by 9 different mammalian species [30], including bats, an ancient mammalian order that is notorious for including reservoir hosts for many re-emerging zoonotic viruses, potentially including SARS-CoV-2 [31]. This evolutionarily conserved role of IRF1 in innate immunity is found also in avian species [32]. This is not unexpected because birds lack several fundamental components of PRR-initiated signaling in mammals, including IRF3, and thus possess a smaller repertoire of innate immune genes [33].

However, there is evidence that IRF1 has continued to evolve functionally since the divergence of vertebrates and invertebrates. For instance, although *Irf1/Irf1-like* transcripts are readily induced by poly(I:C) stimulation in the Pacific oyster, it occurs much more slowly (~48 h after stimulation) [28] than in zebrafish (~12 h) [26] or mammals (as early as 3 h) [14]. Moreover, the IRF1-like protein of the Pacific oyster is expressed within both the nucleus and the cytoplasm, whereas the majority, if not all, of zebrafish and mammalian IRF1 proteins predominantly reside in the nucleus [26,28]. Thus, it seems plausible that IRF1 has continued to evolve to allow more rapid responses to infection by pathogens.

### IRF1 in induced pathogen defense

**Fig 3:**
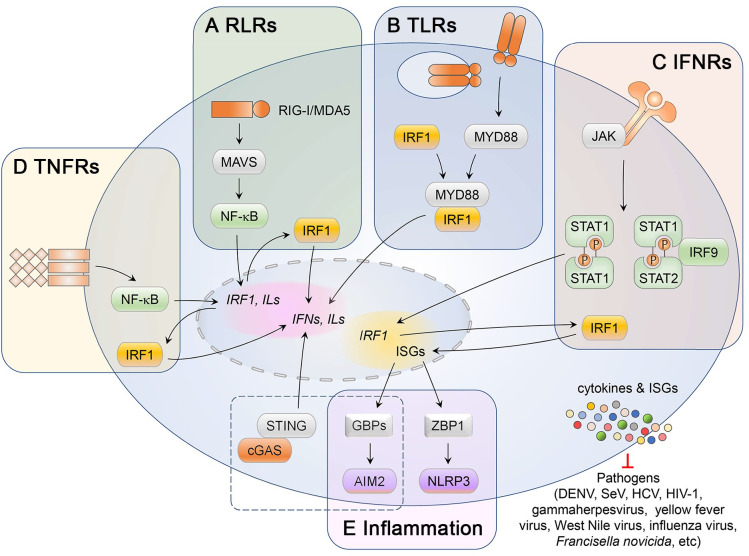
IRF1 in IFN-dependent host defenses. **(A)** RIG-I-like receptors (RLRs) including retinoic acid-inducible gene I (RIG-I) and melanoma differentiation-associated protein 5 (MDA5) associate with mitochondrial antiviral signaling protein (MAVS) to activate the nuclear factor-kB (NF-κB), which induces immediate transcription of *IRF1* mRNA. Newly synthesized IRF1 protein acts downstream to induce interferons and pro-inflammatory cytokines. **(B)** Toll-like receptors (TLRs), except TLR3, interact with the adaptor MYD88 protein, which also interacts with IRF1 so as to license rapid IRF1 migration into the nucleus. **(C)** Interferon receptors (IFNRs) activate Janus kinases (JAKs)- and STAT-dependent signaling cascades, leading to the formation of IFN-stimulated gene factor 3 (ISGF3, a heterotrimeric complex composed of phosphorylated STAT1 and STAT2 with IRF9) and GAF (the STAT1 homodimer complex), which induce IRF1 expression in a positive feedback loop, ultimately augmenting induction of IFN-stimulated genes (ISGs). **(D)** TNF receptors initiate signaling cascades that activate NF-κB, leading to *IRF1* transcription and in turn the expression of type I interferons, but at much lower levels than RLR or TLR signaling pathways. **(E)** Type I IFN-dependent IRF1 expression leads to AIM2- and NLRP3-dependent inflammasome responses to *Francisella novicida* and influenza A virus, respectively. Epigenetic modification including histone methylation and acetylation in the promoter-proximal regions also contributes to dynamic regulation of chromatin states, affecting binding of IRF1 and other transcription factors.

PRRs activate a diversity of signaling pathways upon sensing PAMPs, leading to the transcriptional activation of IFNs and inflammatory cytokines as well as ISGs with a wide range of functions. Similar cascades of signal transduction and transcription are activated by cytokines such as tumor necrosis factor (TNF). Although efforts to elucidate the function of IRF1 extend back almost three decades, the role of IRF1 in innate immune responses remains a developing field. Signaling pathways leading to IFN and/or ISG expression through IRF1 are interrelated and to some extent redundant with signaling via other IRF family members, raising questions as to whether IRF1 is essential for the innate immune defense. In the following subsections, we review IRF1-mediated signaling pathways and how they relate to other signaling pathways that are essential for a balanced IFN response (**Fig 3**).

#### The role of IRF1 in transcriptional responses to RLR engagement

With few exceptions, RNA viruses are sensed by cytoplasmic RIG-I-like receptors (RLRs), including retinoic acid-inducible gene I (RIG-I; also known as DDX58) and melanoma differentiation-associated gene 5 (MDA5; also known as IFIH1). Upon binding viral RNA ligands that serve as PAMPs, RIG-I and MDA5 recruit the mitochondrial antiviral signaling protein (MAVS, also known as CARDIF/VISA/IPS1). This initiates downstream signaling cascades involving multiple kinases that lead to activation of NF-κB and IRFs (in most cases, IRF3). MAVS is expressed on both mitochondrial associated membranes, as well as on peroxisomes, which act similarly as a signaling platform to activate preferentially a type III IFN response [34,35].

As mentioned above, IRF1 is not essential for the induction of type I IFNs [5,6]. Nonetheless, its expression is typically induced rapidly following virus infection or poly(I:C) stimulation [36]. There is clear evidence IRF1 suppresses replication of a variety of RNA viruses [37], and that it plays a key role in host antiviral defense, at least in some cell-type and virus-specific circumstances. For example, IRF1 is important for activating the transcription of type I and/or III IFNs in human hepatocytes that are infected with Sendai virus (a paramyxovirus sensed predominantly by RIG-I) [38] or dengue virus (DENV, a flavivirus sensed by both RIG-I and MDA5) [35]. Similar to human IRF1, murine IRF1 also plays a key antiviral role by activating type I and II IFN responses to DENV infection [37]. Notably, the NF-κB subunits (at least RELA and p50) bind the promoter of *IRF1* and thus drive its transcription [14]. This is reminiscent of the positive IRF7 feedback loop driven by IRF3-induced IFNβ that reinforces IRF3 transcriptional responses to virus infections. A variant of RELA lacking a transactivation domain, designated RELAp43, has been shown to stimulate *IRF1* transcription [39]. However, it is unclear whether RELAp43 activates IRF1 independently of the canonical NF-κB subunits, because it interacts with all NF-κB family members and thus reinforces RELA- and p50-dependent gene expression. Intriguingly, the NF-κB family that mediates *IRF1* transcription has been suggested to share evolutionary features and to have co-evolved with the IRF family [24].

The zebrafish IRF1 protein also induces expression of type I IFNs downstream of RLRs [26]. However, ectopic overexpression of a dominant-negative IRF1 mutant (DBD deletion mutant) has no impact on MAVS-initiated activation of the IFN promoter [26]. This is surprising and, given the crucial role of MAVS in inducing antiviral responses, suggests that IRF1 may not be essential for optimal RLR/MAVS antiviral signaling in fish, as it is in mammalian cells. Alternatively, IRF1 might act independently of MAVS to facilitate RIG-I signaling in early vertebrates.

#### The role of IRF1 in transcriptional responses to TLR engagement

Like RLRs, both plasma membrane-associated and endosomal Toll-like receptors (TLRs) sense PAMPs derived from viruses, and then recruit myeloid differentiation primary-response protein 88 (MYD88) or Toll/Il-1 receptor domain-containing adaptor protein inducing IFNβ (TRIF) to activate IRF3 and NF-κB-dependent expression of type I IFNs. IRF3 and IRF7 have been assumed to be the predominant transcriptional regulators in canonical TLR signaling [40]. However, IRF1 controls TLR9-dependent IFNβ production in mouse myeloid dendritic cells (DC) by interacting with MYD88 [41] (**Fig 3B)**. This interaction with MYD88 enhances the nuclear translocation of newly synthesized IRF1 [42,43].

These findings restored interest in IRF1 as a regulator of type I IFN production. Indeed, IRF1 activation has been found to be associated with TLR7 engagement in conventional DCs [44]. Ectopic expression of MyD88 and IRF1, downstream components in TLR7 signaling, also activates the promoters of both type I and type III IFN genes [45]. In addition, TLR2 activation results in MYD88- and IRF1-dependent IFNβ production [46]. However, an unanswered question in these latter cases is whether IRF1 functions through a specific interaction with MYD88. Analogously, IRF1 is also important for TLR3- and TLR4-induced expression of interleukin 27 p28 (IL27p28) subunit [47], which favors an early type I IFN responsiveness to lymphocytic choriomeningitis virus infection [48]. Zebrafish IRF1 also acts in concert with MYD88 to synergistically activate the promoter of *IFNϕ3* (it is expressed in an *IFNα* fashion) [26]. All of these findings establish IRF1 as an essential component of the TLR-mediated innate immune responses.

#### The role IRF1 in IFN-mediated signaling

Following the recognition of PAMPs by host PRRs and the activation of IRFs, a burst of secretion of multiple types of IFNs triggers Janus kinase (JAK)- and signal transducer and activator of transcription (STAT)-dependent signaling via specific cellular IFN receptors. This paracrine signaling leads to a second wave of transcriptional activation involving the induction of hundreds of ISGs. JAK/STAT signaling induced by the binding of IFNα/β (type I IFN) and IFNλ (type III IFN) to their respective receptors is largely dependent on IFN-stimulated gene factor 3 (ISGF3). ISGF3 is a heterotrimeric complex composed of IRF9 and phosphorylated STAT1 and STAT2 that drives expression of ISGs under transcriptional control of the IFN-stimulated response element (ISRE), an enhancer element with two or more GAAA repeats [49]. By contrast, the binding of IFNγ (type II IFN) to its receptor induces the phosphorylation of STAT1 but not STAT2, leading to the formation of STAT1 homodimers (known as IFN gamma activating factor; *i*.*e*. GAF) that bind to a gamma-activated sequence (GAS, TTCN_[__2__–__4__]_GAA) [50]. The *IRF1* promoter contains a single GAS element rather than an ISGF3-responsive ISRE sequence [51]. Type II IFN thus induces more potent transcription of *IRF1* than either type I or type III IFNs in most cell types [11].

Not surprisingly, the contribution of IRF1 to positive feedback regulation of ISG responses induced by distinct IFNs is complementary, rather than redundant to the role of IRF9 and other IFN-inducible IRFs such as IRF7. This was shown many years ago by northern blot analysis of ISG transcripts in IFNα- or IFNγ-stimulated IRF1 and IRF9 knockout mouse embryonic fibroblasts [52]. More recent, emerging evidence from microarray analysis and chromatin immunoprecipitation sequencing has added significant details to the understanding of IRF1 function by identifying a subset of distinct ISG transcripts driven by IRF1 [12,53]. Through induction of these ISGs, IRF1 contributes to the broad-spectrum innate immune responses to several clinically important viruses, such as gammaherpesvirus, hepatitis C virus (HCV), yellow fever virus, West Nile virus and human immunodeficiency virus type 1 [54,55].

#### The role of IRF1 in TNF-mediated type I IFN signaling

TNF is a pleiotropic cytokine that is closely associated with inflammation, as well as both innate and adaptive immune responses to pathogens. Engagement of its receptors (TNFR1 and TNFR2) triggers distinct signal transduction cascades leading to transcriptional upregulation of selected genes. TNFR1 and TNFR2 differ in their intracellular domains, with TNFR1 having a cytoplasmic ‘death domain’ that is not present in TNFR2. They thus recruit distinct adaptor molecules (TNFR-associated death domain (TRADD) versus TNFR-associated factor (TRAF) 1/2, respectively), although both receptors commonly signal to activate NF-κB [56,57].

TNF may contribute to the induction of IFNβ responses. TNFR1 engagement mediates increased expression of IRF1 and IFNβ, leading to prolonged expression of proinflammatory chemokines via STAT1 [19,58]. Several genes responsive to RIG-I-mediated signaling, including *IRF1*, are similarly induced in human lung epithelial cells stimulated with TNF [59]. These findings suggest that inflammatory responses may favor the activation of an IFN-mediated autocrine loop. This notion is further supported by two independent studies carried out with primary macrophages and murine microvascular endothelial cells [13,60]. TNF signaling contributed to the IFN response to adenoviral vectors, and promoted monocyte recruitment, respectively. Mechanistically, both TNFR1 and TNFR2 (TNFR2 only in human endothelial cells) are required for IRF1-driven, TNF-mediated IFNβ production and autocrine regulation of gene expression. IRF1 responses at early time points were found to depend consistently on NF-κB-mediated *Irf1* transcription in both macrophages and endothelial cells. Whereas macrophages did not require *de novo* synthesis of IRF1 protein for this, endothelial cells required *de novo* protein synthesis. Notably, this latter observation is reminiscent of IRF1-mediated IFN responses triggered by RLRs [14]. However, it should be noted that TNF only induces picomolar concentrations of IFNβ, several orders of magnitude lower than the level of production triggered by RLR or TLR engagement [60]. This weak response may be dispensable for host defenses against pathogens inducing robust IFN responses through other pathways, but may be essential for priming cells for creation of an antiviral state against certain pathogens, including influenza viruses [59].

#### The role of IRF1 in IFN-dependent inflammation

In addition to canonical IFN responses, inflammasome assemblies involving nucleotide-binding domain and leucine rich repeat containing family, pyrin domain containing 3 (NLRP3) and absent in melanoma 2 (AIM2) proteins provide another layer of innate host defense against pathogens. Moreover, type I IFN signaling contributes to both NLRP3 and AIM2 inflammatory responses despite the distinct signals that activate the assembly of these inflammasomes [61–63]. IRF1 has recently been shown to play a pivotal role in the activation of these inflammasomes [64,65]. Mechanistically, infection with the bacterium, *Francisella novicida*, is sensed by the DNA sensor cyclic GMP-AMP synthase (cGAS)—stimulator of IFN genes (STING) pathway that activates type I IFN-dependent expression of IRF1. IRF1 then drives the expression of guanylate-binding proteins (GBPs), leading to intracellular killing of the bacteria and the release of DNA that induces AIM2 activation. Similarly, IRF1 plays an essential role in formation of the NLRP3 inflammasome during influenza A virus infection by inducing transcription of Z-DNA binding protein 1 (*Zbp1*) [65]. By contrast, AIM2-dependent inflammatory responses to the infection with the gamma herpesvirus murine cytomegalovirus (MCMV), or transfection of double-stranded DNA does not require IRF1 [64].

### The contribution of IRF1 to constitutive pathogen defense

**Fig 4:**
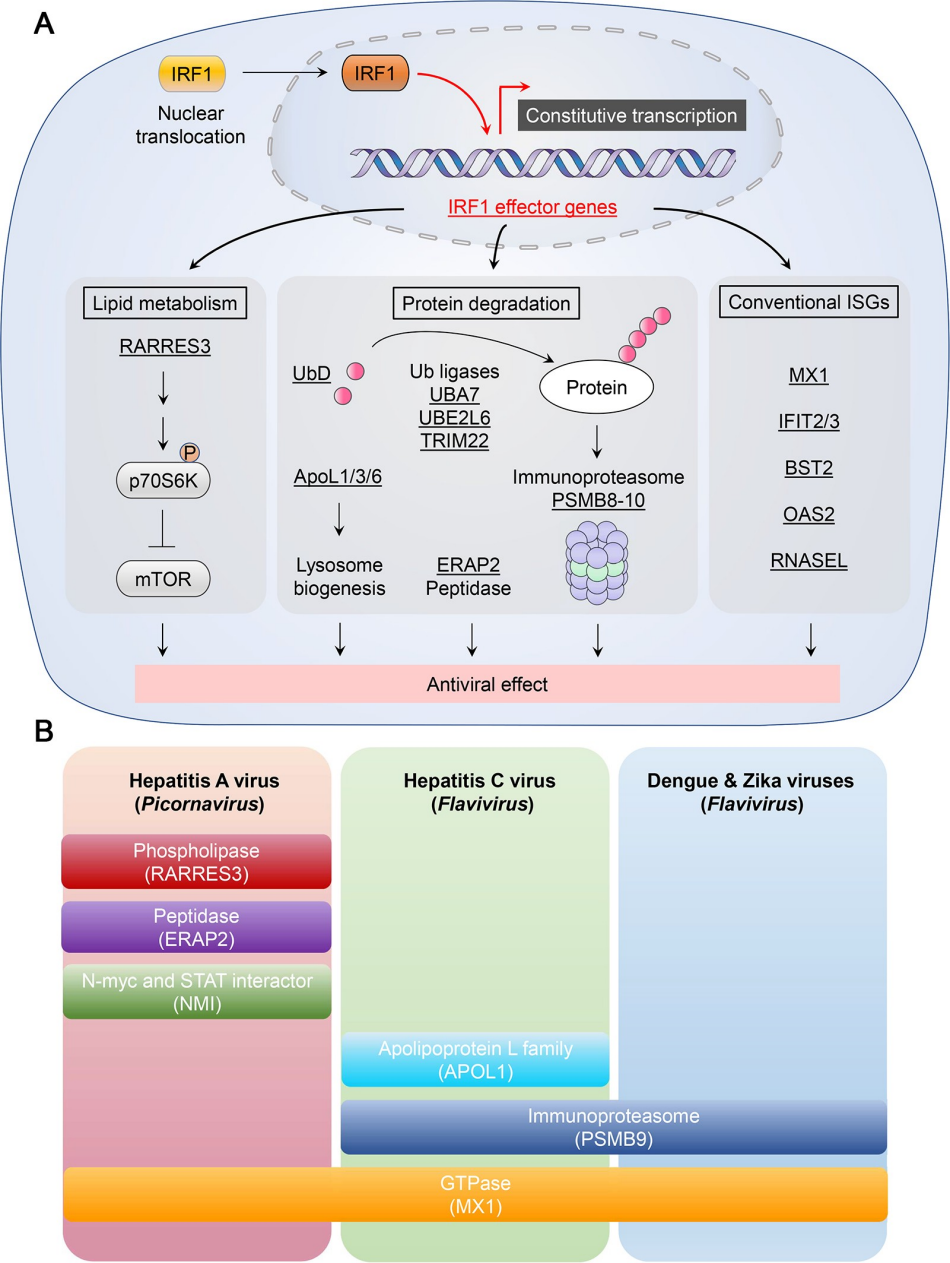
Basal antiviral activity of IRF1 and the downstream effectors. **(A)** Nuclear IRF1 mediates constitutive expression of diverse classes of antiviral effectors acting on lipid metabolism, protein degradation pathways, in addition to driving basal expression of conventional antiviral ISGs such as OAS2. IRF1 target genes are underlined. **(B)** Antiviral spectrum of IRF1 effector genes based on siRNA knockdown assays in immortalized hepatocytes (PH5CH8) infected with hepatitis A virus (HAV), hepatitis C virus (HCV), or flaviviruses (dengue or Zika viruses), or BEAS-2B cells infected with vesicular stomatitis virus.

Most studies examining the role of IRF1 in pathogen defense suggest it is an inducible factor, triggered by infection and contributing to the activation of innate immune responses induced by pathogen sensors such as RLRs, TLRs, or cGAS, as described above. However, a considerable number of studies provide evidence that IRF1 also functions at a basal level, providing constitutive defense against a variety of viruses. Obtaining a quantitative measure of basal antiviral activity is generally challenging, as virus infections are typically associated with unavoidable activation of cell-intrinsic responses via PRRs. However, hepatitis A virus (HAV), an hepatotropic picornavirus, is capable of limited replication in immortalized human hepatocytes without detectable activation of IRF1, or other RLR, TLR, or STING-dependent host antiviral responses [15]. This is consistent with the stealthy nature of (HAV) infection in chimpanzees [66]. Despite the absence of any detectable ‘induced’ antiviral responses, RNA interference (RNAi)-mediated depletion of IRF1 resulted in a 40-fold boost in HAV replication in the immortalized hepatocytes [15]. Notably, the IRF1-mediated restriction of HAV replication was independent of MAVS, IRF3, or STAT1, and persisted in the absence of *de novo* transcription in actinomycin D-treated cells [15]. These results are indicative of a constitutive antiviral state against HAV mediated by IRF1 (**Fig 4A**). A similar phenomenon was noted in human hepatoma cells lacking functional RLR and TLR signaling [15].

High-throughput sequencing of the RNA transcriptome in *IRF1* knockout hepatocytes has provided insight into the role played by basal levels of IRF1 in maintaining constitutive expression of functionally diverse classes of antiviral genes (**Fig 4A**) [15]. The chromatin immunoprecipitation (ChIP)-seq database (publicly available at UCSC Genome Browser https://genome.ucsc.edu) confirms the presence of IRF1-binding motifs in the promoter regions of these genes, validating these genes as *bona fide* targets of IRF1 [67]. Importantly, many of these genes, such as those involved in lipid metabolism (*RARRES3*, *APOL3*, *APOL1*, *OSBPL6*, *APOL6*), have not previously been recognized to possess antiviral activity. A specific combination of these IRF1-regulated genes acts to restrict replication of HAV, whereas other combinations restrict HCV, DENV and Zika virus, all *Flaviviridae* [15]. An exception was myxovirus resistant 1 protein (MX1), which was expressed basally under transcriptional control of IRF1 and broadly restricts replication of these viruses (**Fig 4B**). Another recent study identified 260 genes expressed constitutively in human respiratory epithelial cells under IRF1 control [68]. These included conventional ISGs that contribute to inducible antiviral responses, and have activity against vesicular stomatitis virus (VSV) and influenza virus [68]. While IRF1 effector genes seem vary among different cell types, these studies show that IRF1 can regulate basal antiviral states in various cell types that restrict both positive- and negative-stranded RNA viruses. This is consistent with the lack of an auto-inhibitory domain in IRF1 such as that found in IRF3, -4, -5, and -7 proteins [16], and with observations that IRF1 is predominantly localized within the nucleus in the absence of any exogenous stimuli.

The specific features that render a promoter responsive to basal levels of IRF1 are uncertain, but the environment surrounding the promoter is likely to be important. Basal IRF1 transcriptional activity does not require STAT1, which is often involved in IRF1 transcription triggered by IFNγ or TLR signaling. Recent studies have revealed the existence of IRF1-targeted promoter sites that lack concomitant STAT1 recruitment and are subject to histone acetylation favoring transcription at the basal level [69]. Thus, IRF1-mediated basal transcription may require a distinct set of co-activating factors that differ from those involved in a typical agonist-activated response involving STAT1. Such studies add to our understanding of IRF1 as a constitutively active factor serving a protective role at the basal level [15,68,70].

It is uncertain whether IRF1 orthologs (or IRF11) expressed in fish provide a similar basal level of protection against pathogens. Nevertheless, the existence of IFN homologs exclusively in vertebrates suggests that their evolution has lagged behind that of the IRFs, and that the manner in which the IRFs function in host immunity is likely to be qualitatively different in invertebrates. This does not necessarily imply that the basal protection against virus infections afforded by IRF1 in vertebrates is a vestige of its primordial role in invertebrates. However, it is tempting to speculate that constitutive IRF1 expression may also protect against pathogen invasion in invertebrates, which lack inducible IFN-mediated innate immunity, by regulating genes under control of the IRF-E promoter element.

### IRF1 contributions to transcriptional dynamics and epigenetic gene regulation

#### Cooperative activity with other transcription factors

**Fig 5:**
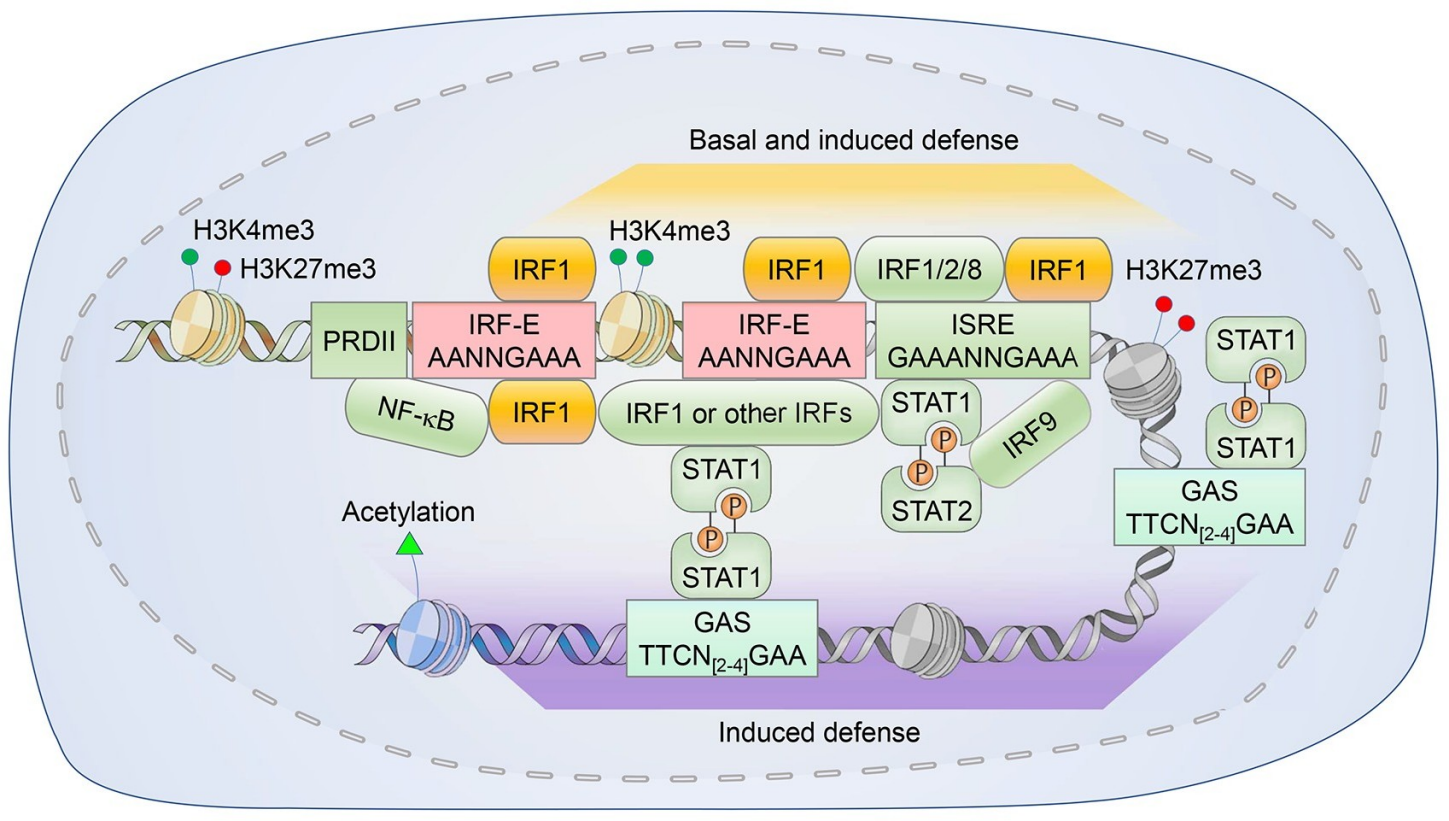
IRF1 contribution to dynamic transcriptional activation networks. IRF1 binds to GAAA sequences within IRF-E and ISRE enhancers, driving transcriptional activation of target genes. In the case of ISRE binding, IRF1 can form homo- or hetero dimers with other members of the IRF family. In genes with multiple *cis*-regulatory elements, coordinate binding of IRF1 and other transcription factors such as NF-κB, ISGF3 or GAF to the enhancer region often has a synergistic impact on gene expression. This can be driven by protein-protein interaction-directed formation of enhanceosomes, or co-localization of transcription factors binding to individual *cis*-elements. IRF1 both modulates and is regulated by activating and repressive epigenetic marks on chromatin, including histone methylation (such as H3K4me3 and H3K27me3) or acetylation at promoter proximal sites.

IRF1 mediates the transcription of a multitude of host defense genes by binding to ‘GAAA’ sites within *cis*-regulatory promoter and enhancer elements. At least two copies of this minimal IRF1 binding site exist within interferon-responsive ISRE elements (Fig 5). IRF1 thus has the potential to bind to either one or both of these GAAA sites as a homodimer, and thus can drive transcription of ISGs. This is particularly important in certain situations, such as when IRF1 is acting at a basal level and exerting its role in constitutive host defenses, or when a functional ISGF3 complex has not been induced by PRR-mediated signaling.

IRF1 can also act cooperatively with other transcription factors to induce gene expression. Distinct sets of genes have been shown to be transcriptionally controlled by IRF1 acting alone versus when it is complexed with IRF8 [71]. It is likely that the transcriptional spectrum is similarly influenced, with different results, when bound to DNA as a heterodimer complexed with other transcription factors, such as IRF2, NF-κB, or STAT1. Hence, the binding of IRF1 to individual IRF-E and ISRE sites in concert with other available transcription factors may dynamically shape the transcriptional status of the genome. These dynamic changes are likely to be particularly prominent as additional transcription factors become activated during the transition from basal, constitutive IRF1-mediated transcription of host defense genes to higher-level responses induced by PRR-initiated signaling. Dimer-specific binding sites exist for IRF3, IRF5, or IRF7 homodimers, and it seems likely that they exist for IRF1 homodimers as well [72].

The presence of multiple *cis*-regulatory elements within promoters and enhancers adds further complexity to the transcriptional activation networks in which IRF1 can engage as part of a protein complex called an ‘enhanceosome’ (Fig 5). For example, the IFNβ promoter contains a PRDII sequence adjacent to its dual PRDIII-PRDI elements, allowing for cooperative binding of IRF1 and NF-κB to a single enhancer region [21]. IRF1 can also act cooperatively with STAT1 to induce ISG expression in IFNγ-treated cells [73]. Cooperative transcriptional activities may be regulated through direct protein-protein interactions involving formation of heterodimers or more complicated multimers [74,75], or *cis*-regulatory element-directed co-localization of transcription factors [76]. Thus, dual binding of IRF1 and other transcriptional factors is not exclusively limited to proximal enhancers [77,78]. Collectively, the existence of a wide variety of IRF1-interacting proteins may account for its functional diversity as well as its role in the cross-talk existing between inflammatory and immune pathways. Theoretically, the expression patterns mediated by engagement of a complex of multiple transcription factors containing IRF1 can be distinguished from that driven by binding of single or homodimeric IRF1. Recent studies have demonstrated the functional importance of cooperative binding of IRF1 and STAT1, but not of either alone, in driving ISG expression following IFNγ stimulation in multiple cell types [77].

#### Modification of the epigenetic landscape

Epigenetic modifications dynamically regulate the state of chromatin, thereby influencing the binding of IRF1 and its cofactors to promoter elements. They thus contribute to the complexity of gene expression during the host response to infection, as well as in the pathogenesis of autoimmune diseases (Fig 5). For instance, changes in histone methylation status to active (H3K4me3) or repressive (H3K27me3) marks at IRF1-binding sites modulate transcription of IRF1-target genes [79,80]. Histone acetylation also promotes transcriptional activation by providing a more permissive state for the binding of transcription factors [81]. Importantly, chromatin enrichment in histone active marks is implicated in the pathogenesis of systemic lupus erythematosus (SLE), an autoimmune disease associated with increased type I IFN responses. IRF1 binding sites are robustly enriched in histone H4 acetylation or H3K4me3 methylation in monocytes from SLE patients [82,83]. IFNα stimulation promotes the direct association of IRF1 with p300, a protein lysine acetyltransferase that mediates the addition of acetyl groups to histone lysine residues, to favor pathologic H4 acetylation [82]. Thus, in addition to its transcriptional activity being influenced by histone marks on the chromatin, IRF1 shapes how the chromatin is marked epigenetically through direct interactions with histone-modifying enzymes [84].

Chromatin immunoprecipitation (ChIP) analyses suggest that the switching of IRF1 activity from a basal to an agonist-induced state may be regulated in part epigenetically at the level of histone acetylation [69]. In the basal state, histone acetylation is biased toward H3 and tends to be isolated at IRF1 binding sites, whereas IFNγ stimulation causes acetylation toward H4 and preferentially generates dual H3-H4 acetylation sites, thereby causing a shift from basal IRF1 binding toward an induced dual binding of IRF1 and STAT1 [69]. Of note, only a quarter of IRF1 sites undergo such a shift. The majority of IRF1 binding sites fail to recruit STAT1 and show either constitutive (31%) or no histone acetylation (44%), with a minority being marked with repressive methylation (e.g. H3K27me3). Thus, the basal activity of IRF1 is regulated by constitutive histone acetylation, whereas agonist-induced IRF1 transcription depends on the recruitment of additional transcription factors (STAT1, for example) and histone acetyltransferases [69]. What controls these differences in the epigenetic modification of these sites is uncertain. IRF1 binding at ‘nonproductive’ sites marked by H3K27me3 that fail to undergo IFNγ-induced acetylation remains dependent upon the chromatin-modifying enzyme SMARCA4 (SWI/SNF-related, matrix-associated, actin-dependent regulator of chromatin, subfamily A, member 4) [69]. These ‘nonproductive’ IRF1 binding sites are often distant from promoters, but nonetheless likely contribute to basal, IRF1-dependent gene expression. Adding further to the complexity of IRF1 associated epigenetic regulation is evidence that interleukin 4 (IL-4) can directly inhibit epigenetic and transcriptional changes induced by IFNγ near binding sites for the auxiliary transcription factor AP-1 [85]. Such IRF1-dependent epigenomic and transcriptional cross-talk likely ensures a robust yet carefully balanced execution of host immune responses to a variety of stimuli.

### Conflicts at the IRF1 line of defense

**Fig 6:**
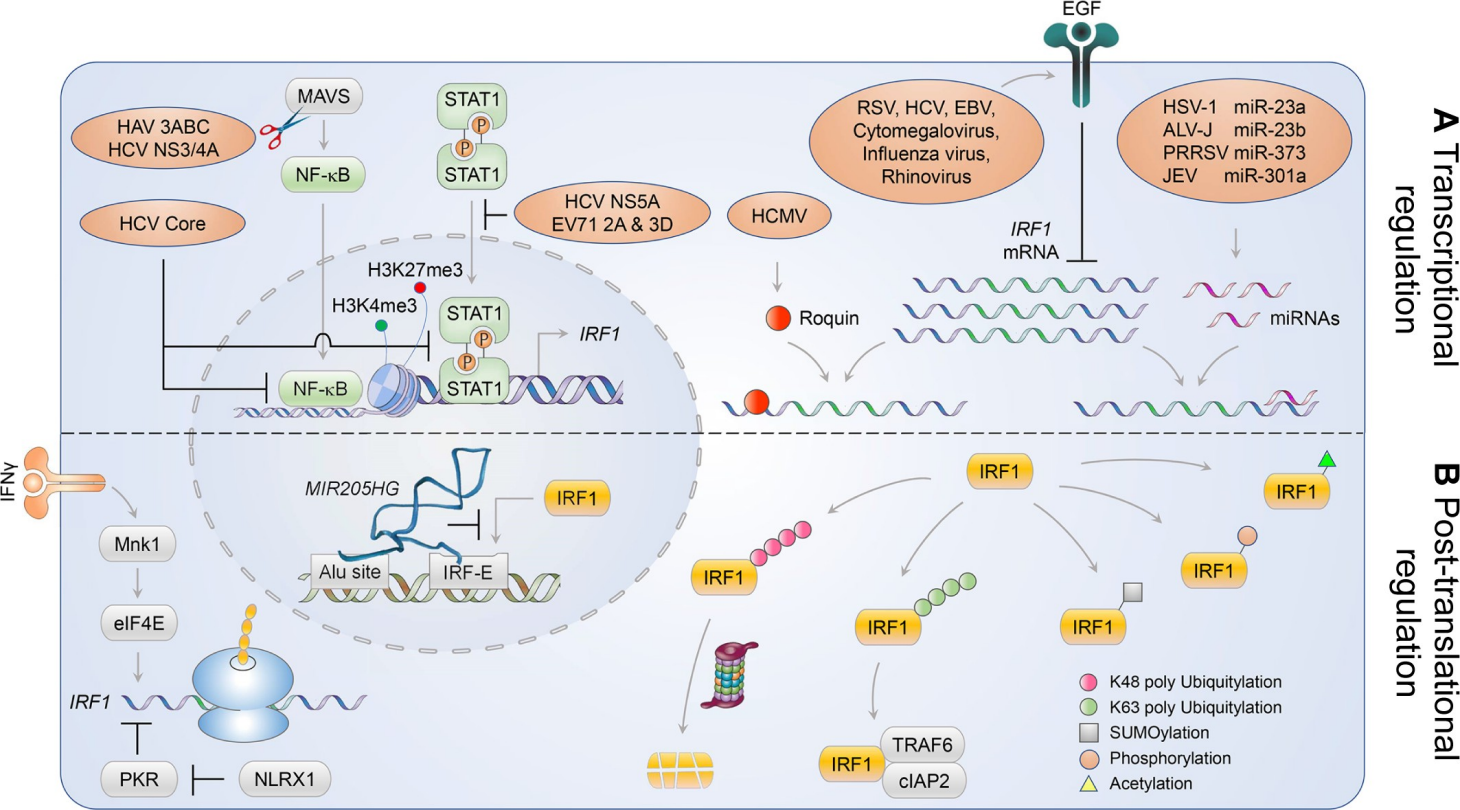
Regulation of IRF1 responses by virus and host. Various pathways cross-regulate IRF1 responses, altering the expression level and activation state of IRF1. **(A)** Transcription of *IRF1* is mediated largely by NF-κB and GAF, which are antagonized by HCV core protein. *IRF1* transcription is also is regulated epigenetically by histone H3 methylation and acetylation. While both HAV and HCV encode proteases that disable MAVS signaling, HCV and EV71 disrupt the nuclear translocation of GAF. HCMV usurps the host Roquin protein to reduce *IRF1* mRNA levels by binding to the 5′ UTR of *IRF1* transcripts, while other viruses activate EGFR signaling to downregulate *IRF1* mRNA. miRNAs also mediate downregulation of *IRF1* mRNA levels. **(B)** IFNγ receptor signaling activates Mnk1/eIF4E to ensure translation of *IRF1* mRNA, whereas NLRX1 promotes IRF1 protein synthesis by counteracting PKR-mediated global protein translation. The host gene encoding miR-205 associates with the promoters of its target genes at Alu sequences in proximity of IRF-E motif, thereby suppressing IRF1 binding. Activity of IRF1 protein is also regulated post-translationally via ubiquitination, SUMOylation, phosphorylation and acetylation.

Phylogenetic analyses of the IRF proteins suggest that IRF1 represents a crucial component of host defense against pathogen invasion. Indeed, the ability to govern events at the IRF1 line of defense contributes substantially to the outcome of pathogenic infections. Thus, both host and pathogen have evolved strategies to control IRF1 signaling in an effort to gain the upper hand in this conflict. In this section we focus on the diverse mechanisms that regulate IRF1 activity at the mRNA transcript and protein levels (**Fig 6**).

#### Regulation of IRF1 at the transcript level

As discussed above, increases in IRF1 expression induced by infection primarily result from NF-κB and STAT1-mediated transcriptional activation. It is not surprising, therefore, that numerous viruses have evolved countering strategies that target upstream components of NF-κB- and STAT1 signaling that can disrupt *IRF1* transcription and mitigate downstream antiviral responses [86]. HCV, a positive-strand RNA virus in the *Flaviviridae* family, provides a prime example of this. HCV expresses a protease, NS3/4A, that degrades the adaptor protein MAVS to blunt RLR signaling, as does the phylogenetically distinct picornavirus, HAV [87,88]. In addition, the HCV core protein interacts with the *IRF1* promoter to repress IRF1 synthesis at the level of transcription [89]. If that were not enough to disable IRF1, the NS5A protein of HCV suppresses *IRF1* expression by modulating STAT1 phosphorylation to ablate formation of STAT1 homodimers known as the IFNγ-activated factor, GAF [90]. Likewise, the 2A and 3D proteins of enterovirus D71 dampen nuclear translocation of STAT1, sequestering it from the *IRF1* promoter in IFNγ-stimulated cells [91].

Airway infections with rhinovirus or respiratory syncytial virus (RSV), RNA viruses with distinctly different genome structures and replication strategies, can result in oxidative tissue damage leading to an epithelial-mesenchymal transition (EMT), thereby silencing *IRF1* transcription [36]. Mechanistically, EMT is associated with induction of zinc finger E-box binding homeobox 1 (ZEB1) expression, leading to the formation of ZEB1 C-terminal binding protein (CtBP) polycomb repressive complex (PRC) 2 that forms repressive H3K27me3 histone marks on the *IRF1* promoter. Mesenchymal transitioned cells thus become epigentically defective in inducible *IRF1* expression due to occluded access of transcription factors, including RelA [36]. Respiratory virus infections can also suppress *IRF1* responses by activating epidermal growth factor receptor (EGFR) signaling, although mechanistic details are lacking [92]. Alternatively, viruses can usurp cellular components to downregulate *IRF1* expression and thus inhibit IRF1-mediated innate immune responses. For instance, human cytomegalovirus (HCMV) has recently been shown to induce the expression of roquin, an RNA-binding protein that interacts with a stem-loop in the 5’ untranslated region (5’UTR) of *IRF1* transcripts to inhibit their translation [93].

Viruses also modulate the expression of microRNAs (miRNAs) that target the 3´UTRs of *IRF1* transcripts to downregulate IRF1 expression. miR-301a is transcriptionally induced by Japanese encephalitis virus (a neurotropic virus of the *Flaviviridae* family) through NF-κB, leading to reduced expression of IRF1 [94]. An integrative analysis of the miRNome and transcriptome confirmed an inverse correlation between miR-301a-3p and its target gene *IRF1* transcript at late time points following IFNγ stimulation [95]. Whereas some miRNAs facilitate virus replication by suppressing *IRF1* expression, other miRNAs can potentiate intrinsic innate immune responses via IRF1. One such example is the liver-specific miR-122, which targets receptor tyrosine kinases leading to reduced phosphorylation of STAT3 at Tyr705, thereby counteracting the negative impact of STAT3 on IFN signaling by facilitating *IRF1* expression [96]. Long noncoding RNAs (lncRNAs) may also modulate the expression of IRF1, although their subversion by viruses has yet to be documented. MIR205HG, a nuclear lncRNA from which miR-205 is derived, associates with Alu sequences in proximity to IRF binding sites, negatively influencing the transcriptional activity of IRF1 (and possibly other IRFs) [97].

#### Regulation of IRF1 at the protein level

Despite the predominant transcriptional regulation of IRF1, IRF1 responses are also regulated at the level of protein synthesis. IFNγ stimulation rapidly activates MAPK-interacting protein kinase 1 (Mnk1) and its downstream target, eIF4E, in a Mek/Erk-dependent manner, thereby ensuring translation of IFN-induced *IRF1* mRNA [98]. Surprisingly, the nucleotide-binding oligomerization domain-like receptor (NLR) protein NLRX1, a well-documented negative regulator of innate immunity [99], is essential for virus-stimulated *de novo* synthesis of IRF1 protein [14]. Mechanistically, NLRX1 competes with the innate immune sensor, protein kinase R (PKR), for binding viral double-stranded RNA replication intermediates and poly(I:C). This protects the translation of *IRF1* mRNA from PKR-mediated global shutdown of cellular protein synthesis, and is crucial for RLR-initiated cytokine responses in immortalized primary adult human hepatocytes [14].

Post-translational modifications also add a variety of functionalities to IRF1. In particular, the turnover and transcriptional activity of IRF1 is tightly regulated by K48-linked ubiquitination of the 39-residue C-terminal region of IRF1, which leads to its degradation via the proteasome [100]. Viruses have evolved mechanisms that take advantage of this ubiquitin-proteasome pathway to destroy IRF1 and evade the host immune response [101]. While degradation of IRF1 is regulated in response to dynamic cellular conditions and specific stresses [95], its ubiquitin-mediated turnover is coupled with optimal transcription activity [102]. Glycogen synthase kinase 3β (GSK3β)-mediated phosphorylation of IRF1 at Thr181/Ser185 allows for Skp1-Cullin1-F-box/F-box and WD repeat domain-containing 7α (SCF/Fbxw7α)-mediated K48-linked polyubiquitination and subsequent proteasomal degradation, thereby facilitating the removal of unused IRF1 molecules accumulating at target promoters. In contrast to K48-linked ubiquitination that targets IRF1 for degradation, K63-linked ubiquitination contributes to its activation. Notably, K63-linked polyubiquitination of IRF1 is mediated by recruitment of TRAF6 and cellular inhibitor of apoptosis 2 (cIAP2), and is essential for activation of newly synthesized IRF1 protein in response to interleukin 1 (IL-1) stimulation [103] and TLR7/8 engagement [104].

SUMOylation also modifies IRF1 activity to suppress its transcriptional activity. This occurs at sites that are identical to the major ubiquitination sites within the C terminus of IRF1, enhancing its resistance to degradation via the ubiquitin-proteasome pathway [105]. K48-linked ubiquitin-mediated degradation and K63-linked ubiquitin optimization of IRF1 activity are inherently tied to each other, and stabilization by SUMOylation does not necessarily result in upregulation of overall IRF1 activity. Phosphorylation and acetylation of IRF1 have also been reported. However, the functional significance of IRF1 phosphorylation remains debated [106]. Studies with zebrafish IRF1 did not show a need for phosphorylation for its DNA binding activity [26]. By contrast, IRF1 acetylation has been proposed to be important for activation of the long terminal repeat promoter of HIV-1 in the absence of the viral transactivator, Tat [107].

### IRF1 functions outside the realm of host-pathogen interactions

In addition to protection from infection by pathogens, IRF1 is recognized to be a key regulator of tumor progression and to have tumor suppressor properties. Genetic deletions of either or both alleles of IRF1 located on chromosome 5q are frequently detected in human leukemia and myelodysplasia [108]. Likewise, heterozygous loss of IRF1 is common in breast cancer and esophageal and gastric carcinomas. In parallel with these clinical observations, experimental data suggest IRF1 is able to counteract the pro-oncogenic activity of IRF2 or activated oncogenes [109,110]. Although later studies with mice suggested that IRF1 deficiency by itself does not lead to spontaneous tumor development, loss of IRF1 clearly promotes tumorigenesis in mice with a p53 null genetic background or Ha-ras expression [111]. IRF1 has both anti-proliferative and transformation-suppressive properties, and is also pro-apoptotic [110,112]. Intriguingly, IFNs themselves negatively regulate proliferation in many cell types, and are capable of eliciting anti-tumor immunity [113].

Similar to the antagonism of host innate immunity by pathogens, tumors evolve to escape from anti-tumor immune surveillance. A notable example is programmed cell death 1 ligand 1 (PD-L1), a ligand of the programmed cell-death protein 1 (PD-1) immune inhibitory checkpoint that is induced in tumors upon IFN exposure and leads to immune evasion [114]. The expression of PD-L1 is regulated by the IFNγ/STAT1/IRF1 axis in melanoma cells; IRF1 binds directly to the PD-L1 promoter [115]. Changes in IRF1 expression thus affect the dynamic balance between host anti-tumor immunity and tumor escape by regulating PD-L1 expression [116,117]. IRF1 regulates both basal and IFNγ-induced expression of PD-L1 [118]. IRF1-induced PD-L1 expression may favor the progression of certain types of tumors, such as malignant melanoma, but basal PD-L1 levels may be sufficient to blunt host anti-tumor immunity. IRF1 may thus have opposing effects on different tumors, promoting progression of PD-L1-dependent tumors, and restricting others in which PD-L1-independent mechanisms account for tumor proliferation.

Although an ortholog of protein kinase R (PKR), an IRF1-regulated, IFN-inducible protein with tumor-suppressor activity, exists in zebrafish [119], the tumor-suppressor potential of IRF1 remains uncertain among lower vertebrates.

### Closing remarks

Research over the past 30 years has revealed in fine molecular detail how IRF1 is regulated and the roles it plays in maintaining host homeostasis. Among other housekeeping activities exemplified by its role in tumor suppression, IRF1 protects the host from invading pathogens at multiple levels, ranging from an intrinsic ‘basal’ level to a robust ‘inducible’ function. IRF1 thus acts as a master-regulator of innate immunity by regulating antiviral status at a basal, steady state, and also by orchestrating multiple cellular signaling cascades that effect a wide spectrum of antiviral responses that range from alterations in lipid metabolism to protein degradation machineries. The fundamental role of IRF1 has been conserved throughout evolution. Only recently, however, have we begun to appreciate how competition between host and pathogen, coupled with the emergence of the interferon system in vertebrates, has shaped the role of IRF1.

Several important gaps exist in our mechanistic understanding of IRF1 function. For instance, it is entirely unclear how the basal activity and nuclear localization of IRF1 are maintained. Are there upstream components that post-translationally modify the IRF1 molecule via ubiquitination or phosphorylation in a fashion analogous to an agonist-activated state? Do the profiles of IRF1 target genes similar in different cell types? Is there therapeutic potential in manipulating IRF1-responsive pathways, a possibility that has yet to be explored? Therapies that augment IRF1 activity could be beneficial in defeating a broad range of pathogens, but they could also pose serious threats to the host if uncontrolled immune activation led to autoimmune-like disease. Drugs that specifically boost individual IRF1 effector functions (such as the phospolipase *RARRES3*) might provide narrower, yet equally effective and safer host-directed therapeutic approaches. Given the diverse array of effectors that exist in the IRF1 regulome, understanding the mechanisms underlying the protective role of IRF1 can be expected to provide key insights into microbial infections and host defense, as well as cancer biology where IRF1 also plays a pivotal role in disease outcome.
